# Clinical efficacy analysis of mucoperiosteal flap combined with perforation technique for tooth extraction in medication-related osteonecrosis of the jaw risk population: a retrospective study

**DOI:** 10.1186/s12903-025-07130-8

**Published:** 2025-11-04

**Authors:** Ying Zhou, Qingxiang Li, Hongyuan Huang, Qiao Qiao, Jing Wang, Na Ge, Enbo Wang, Jianhua Zhu, Yuxing Guo

**Affiliations:** 1 Department of Oral and Maxillofacial Surgery, Peking University School and Hospital of Stomatology, No.22 Zhong Guan Cun South Ave, Beijing, 100081 China; 2 National Center of Stomatology, National Clinical Research Center for Oral Diseases, National Engineering Research Center of Oral Biomaterials and Digital Medical Devices, Beijing Key Laboratory of Digital Stomatology, NHC Research Center of Engineering and Technology for Computerized Dentistry, NMPA Key Laboratory for Dental Materials, Peking University School and Hospital of Stomatology, No.22 Zhong Guan Cun South Ave, Beijing, 100081 China

**Keywords:** Medication-related osteonecrosis of the jaw, Mucoperiosteal flap, Perforation technique, Tooth extraction, Anti-resorptive drugs

## Abstract

**Background:**

To analyze and summarize the clinical efficacy of mucoperiosteal flap combined with perforation technique for tooth extraction in medication-related osteonecrosis of the jaw risk (MRONJ) population, and to provide clinical treatment options for such patients.

**Methods:**

This study included 51 patients receiving antiresorptive drug (ARD) treatment who underwent tooth extraction at the Department of Oral and Maxillofacial Surgery, Peking University School and Hospital of Stomatology between November 2016 and February 2024. Preoperative clinical data were collected and analyzed. Based on their drug history, patients were categorized into two groups: (1) low-dose ARD group for patients with non-neoplastic lesion like osteoporosis (LDA), (2) high-dose ARD group for patients with bone metastatic lesions like breast cancer (HDA). All patients underwent tooth extraction using mucoperiosteal flap combined with perforation technique. Follow-up assessments were conducted at 1 week, 2 weeks, 1 month, 3 month and 6 months postoperatively. The primary evaluation outcome was whether the patient had developed MRONJ at the extraction site at 6 months. The secondary evaluation outcome was whether the extraction socket mucosa healed completely within 1 month. Statistical analysis included Chi-square, Fisher’s exact, Mann–Whitney U/t tests and survival analysis to contrast between LDA and HDA group. Significance was set at *p* ≤ 0.05.

**Results:**

A total of 82 extraction sites in 51 patients (31 females in LDA group with a mean age of 66.58 ± 13.29 years. 14 females and 6 males in HDA group with a mean age of 58.24 ± 11.63 years) were included in the study. Of these, 47 extraction sites in the LDA group, 35 extraction sites in the HDA group. During postoperative follow-up, MRONJ only occurred in 2 extraction sites in HDA group in two patients, resulting in a postoperative clinical healing rate of 100% (47/47) in LDA group and 94.3% (33/35) in HDA group. A significant difference in the time to extraction socket mucosal healing was observed between the LDA and HDA groups (*p* ≤ 0.05).

**Conclusions:**

This study demonstrated that in patients with potential risks of MRONJ, the application of mucoperiosteal flap combined with perforation technique based teeth extraction method could safely and effectively alleviate the dental inflammation in the oral cavity, and mitigates the risk of MRONJ development.

## Background

MRONJ represents a rare yet severe complication characterized by pathologically accelerated osteocyte apoptosis and osteoblast dysfunction, induced by prolonged exposure to ARDs, antiangiogenic agents, or steroids [1, 2]. These agents are frequently employed in clinical practice for managing bone metastasis associated with malignant tumors, as well as for treating osteoporosis and other skeletal disorders [3]. Patients with MRONJ may present with symptoms such as exposed jawbone, soft tissue inflammation, severe pain, and jawbone fractures, all of which significantly affect the quality of life [4]. Studies have reported that between 58% and 86% of patients who develop MRONJ have undergone tooth extraction [5, 6]. The chronic inflammatory state associated with advanced periodontitis or apical periodontitis can facilitate bacterial penetration into the gingival sulcus or root canal via the periodontal pocket [7]. This process disrupts altered bone turnover dynamics, leading to localized increases in bone mineral density, compromised intraosseous microcirculation, and subsequent osteoblast apoptosis, and ultimately osteonecrosis of the jaw, potentially progressing to MRONJ [8–10]. A retrospective cohort study by Avishai et al. analyzed 103 extraction sites in 93 patients and found that when the indication for extraction was periodontal disease, longitudinal root fissures, or periapical lesions, the odds of MRONJ after extraction increased 4.29 times [11]. The Italian Consensus Update on MRONJ prevention and diagnosis recommends that when conservative treatment of an infected tooth of odontogenic origin is no longer effective and prognosis is uncertain, the tooth should be extracted promptly to resolve the inflammatory process and reduce the risk of MRONJ [12].

Building upon principles derived from cortical perforation techniques utilized in osseous reconstruction for enhanced vascularity during implantology procedures, our research group has developed an innovative modified jaw curettage protocol designed to mitigate MRONJ progression in susceptible patients [13]. This modified approach, applied clinically, demonstrated significantly better outcomes than the traditional technique [14]. Building on these discoveries, our group has proposed an innovative approach “mucoperiosteal flap combined with perforation technique” for tooth extraction. This strategy was evaluated through initial small-scale clinical trials, demonstrating its efficacy and safety [15]. In this report, we provide a comprehensive analysis of the clinical outcomes achieved using this treatment modality in a moderately sized patient sample.We have specifically expanded the cohort of patients on HDA group and introduced “Time since the last administration of ARD (months)” as a key analytical variable. Additionally, the follow-up period has been extended from 3 to 6 months, with the incorporation of soft tissue healing status at 1 month post-operation as a new endpoint.

## Methods

### Study design and settings

Prior to initiating this retrospective study, we sought approval from the Biomedical Ethics Committee of Peking University School and Hospital of Stomatology. (Approval No. PKUSSIRB-201949113). This investigation was conducted among patients at risk of MRONJ, who were comprehensively informed about the study’s objectives and related procedures. Each participant provided written, signed, and dated informed consent prior to commencing any treatments.

### Participant and clinical data selection

Patients who underwent tooth extraction at the Department of Oral and Maxillofacial Surgery, Peking University School and Hospital of Stomatology from November 2016 to February 2024 were included in this study. The inclusion criteria comprised patients who (1) had history of anti-resorptive drug use; (2) presence of affected teeth that are deemed non-salvageable, such as those with chronic periodontitis, apical periodontitis, residual crowns or roots, fractured teeth; (3) willingness to undergo extraction using the mucoperiosteal flap combined with perforation technique and sign an informed consent form; (4) availability of complete clinical data and a follow-up period of at least 6 months. Exclusion criteria included patients with (1) prior head and neck radiation, (2) prior MRONJ at or close to the extraction site, and (3) history of tooth extraction in the same quadrant within 3 months before enrollment. All enrolled patients were required to have discontinued the medication for at least 1 month prior to tooth extraction. Clinical data were collected for all patients, including gender, age, primary disease, medication regimen, duration of medication use, systemic condition, and indication for tooth extraction and so on. The primary evaluation outcome was whether the patient had developed Medication-related osteonecrosis of the jaw (MRONJ) at the extraction site at 6 months. And the secondary evaluation outcome was whether the extraction socket mucosa healed completely(defined as complete mucosal coverage and no clinical signs of infection or inflammation) within 1 month [16].

### Classification of patients

Based on the primary disease and the characteristics of the antiresorptive drug history, the patients were classified into two groups (Table 1).

**Table 1:** Classification of patients

Classification	Patient characteristics
Low-dose anti-resorptive drug group (LDA)	Patients with non-neoplastic disease (mostly patients with primary or secondary osteoporosis, or those who are preventing osteoporosis) are treated with oral or intravenous medications. Common drugs include alendronate, etc.
High-dose anti-resorptive drug group (HDA)	Patients with bone metastatic lesions of malignant tumors or giant cell tumors of bone treated by intravenous or subcutaneous administration; common drugs include zoledronic Acid and Desutumab.

### Surgical treatment process

**Fig. 1:**
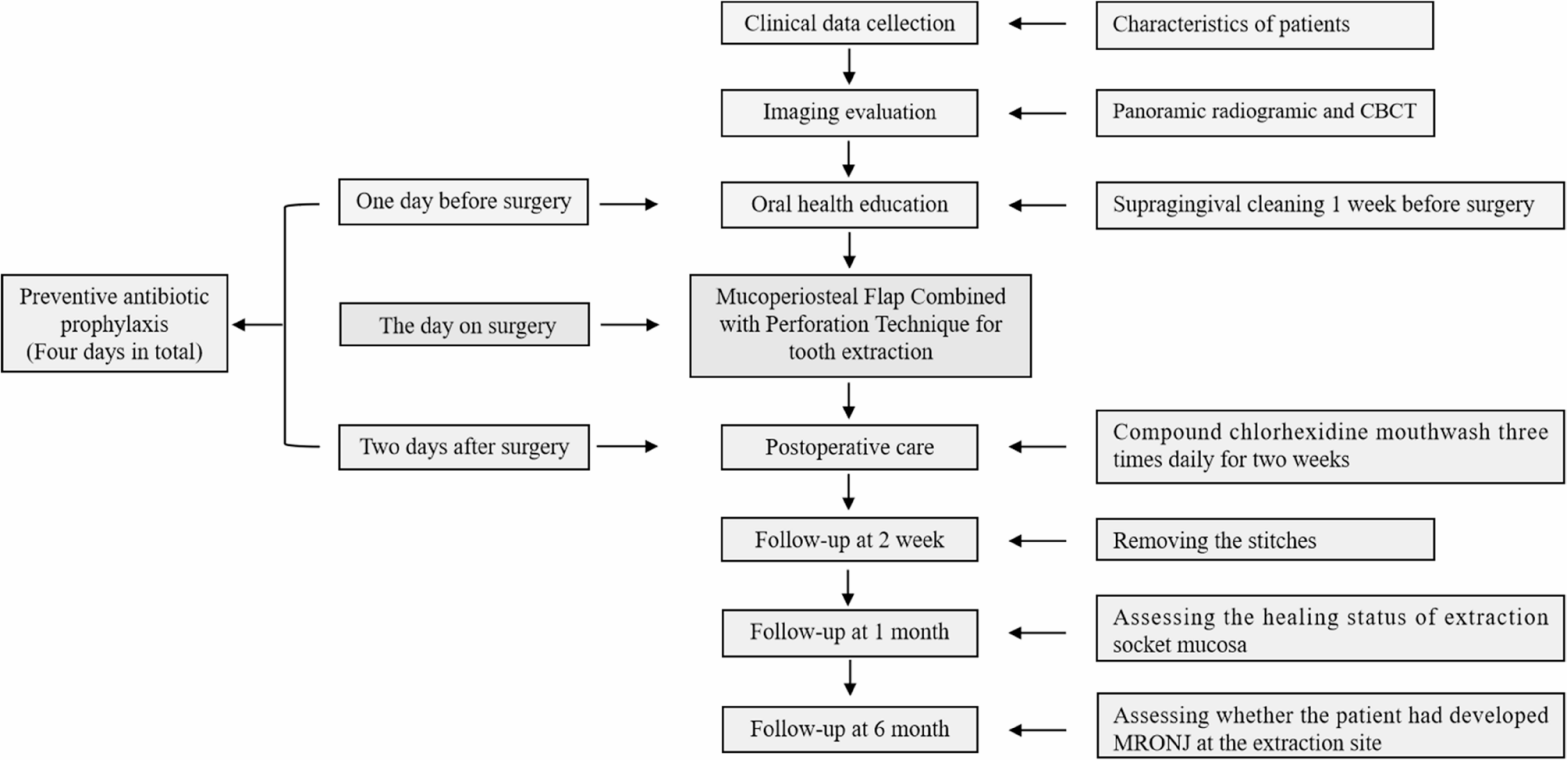
Surgical treatment process for tooth extraction of mucoperiosteal flap combined with perforation technique

The surgical treatment process is demonstrated in Fig. 1 with the following details. Imaging evaluation, including panoramic radiograhy and cone beam computed tomography (CBCT), was completed preoperatively to rule out jaw pathology, such as osteonecrosis of the jaw, cysts, and metastases. Periodontal scaling of teeth in the mouth was completed one week prior to surgery, except for focal teeth. Prophylactic antibiotics were administered from 1 day before to 2 days after the operation (4 days in total) [17]. The antibiotic regimen consisted of amoxicillin-clavulanate potassium; for patients with penicillin allergies, azithromycin was used as an alternative. Patients were instructed to rinse the mouth gently with compound chlorhexidine mouthwash three times daily for two weeks. For the first two days, semi-runny warm and cool food was recommended, followed by a gradual return to normal food. Stitches were removed two weeks after the surgery. Patients were followed up at 1 week, 2 weeks, 1 month, and 6 months post-surgery. The primary outcome measure was the occurrence of medication-related osteonecrosis of the jaw (MRONJ) at the extraction site 6 months after the procedure. Diagnostic criteria for MRONJ were based on the guidelines published by the American Association of Oral and Maxillofacial Surgeons (AAOMS) in 2022 [1].

### Mucoperiosteal flap combined with perforation technique

**Fig. 2:**
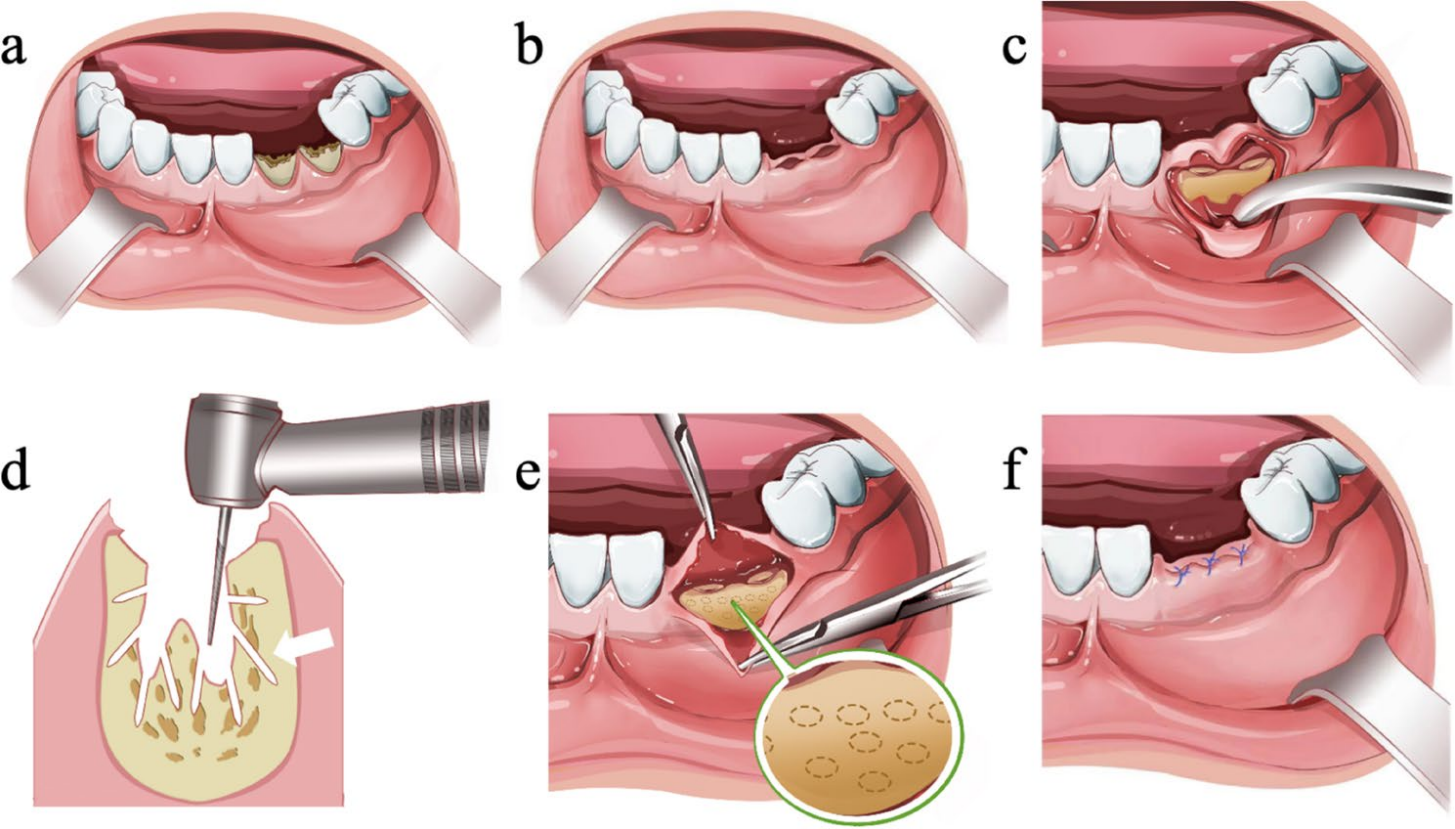
Mucoperiosteal flap combined with perforation technique for teeth extraction. Tooth was extracted after local anesthesia (**a**, **b**),and then trapezoidal or angular flap was performed in the proximal and distal mesial gingival papilla, and the flap was raised under the periosteum (**c**), the extraction socket was thoroughly scratched, and the bone perforations around the extraction wounds pointed out by white arrows (**d**) were prepared with a fine fissure bur under the cooling of sterile saline solution with the density of the perforations spaced at 1 mm intervals at a depth of 2–3 mm(the enlarged circular dashed line represents perforations around the extraction socket) (**e**) the mucoperiosteal flap was advanced to be completely closed, and the periosteal flap was loosened or the alveolar ridge was appropriately lowered for decompensation if tension existed; the wounds were closed by interrupted suturing of 4/0 absorbable suture material (**f**)

Anesthesia was administered based on the tooth position, either via block or local infiltration. A trapezoidal or angular incision was made at the proximal and distal mesial gingival papillae of the affected teeth, followed by a subperiosteal flap raising. Teeth were extracted using extraction forceps, dental tappets, or a turbo-drill with minimal invasiveness. After extraction, the sockets were thoroughly debrided, and a fine fissure bur was used to prepare the cortical bone perforation under sterile saline solution cooling. Bone perforation density intervals were set at 1 mm, with a depth of 2–3 mm, while the roots of neighboring teeth and the inferior alveolar nerve need to be avoided. The complete closure of the wound was achieved by relieving the tension of the mucoperiosteal flap or the alveolar ridge was appropriately lowered. The wound was closed using intermittent sutures with 4/0 absorbable suture material (Vicryl Rapide™ (Polyglactin 910) suture (Ethicon Inc, Somerville, NJ, USA).(Fig. 2).

### Statisticl analysis

All statistical analysis were conducted using IBM SPSS Statistics(version 27.0 for windows). The collected data were categorized based on patient characteristics and extraction sites and assigned to LDA and HDA groups, as detailed in Tables 1 and 2. Descriptive statistics were computed by determining the mean values, standard deviations (SD), and cumulative frequencies of the respective outcomes. Statistical analysis included Chi-square, Fisher’s exact, Mann–Whitney U tests to contrast between LDA and HDA group.The Generalized Estimating Equations (GEE) was employed to analyze the relationship between different groups and the time to healing. Statistical significance was determined at *p* ≤ 0.05.

**Table 2:** Data at a patient level for LDA and HDA groups

Characteristic		LDA	HDA	*p*-value
Number of patients		31	20	
Age(years) (mean ± SD)	Mean ± SD*	66.58 ± 13.29	58.24 ± 11.63	*0.013*
Sex, *n*	Female	31	14	*0.001*
	Male	0	6	
Primary disease, *n*	Osteoporosis	30	0	*< 0.001*
	Osteogenesis imperfecta	1	0	
	Breast cancer	0	12	
	Lung cancer	0	3	
	Multiple myeloma	0	2	
	Prostate cancer	0	1	
	Neuroendocrine cancer	0	1	
	Uterine cancer	0	1	
Systemic disease, *n*	Diabetes	7	3	0.777
	Rheumatoid	2	0	
	Hypertension	5	2	
	Others	2	1	
	None	15	14	
Specific ARD used, *n*	Zoledronic Acid	2	15	*< 0.001*
	Denosumab	4	4	
	Alendronate	25	0	
	Ibandronate	0	1	
Time on ARD (dose)	Mean ± SD*	3.04 ± 5.08	23.16 ± 5.08	*< 0.001*
Time since last administration of ARD (months)	Mean ± SD*	7.8 ± 4.58	14.9 ± 3.82	0.132

The *p*-values correspond to the outcomes of the Chi-square/Fishe’s exact test for comparing LDA and HDA group. Variables marked with an asterisk (*) indicate ordinal/numerical data analyzed using the Mann–Whitney U test. Significant *p*-values (*p* ≤ 0.05) are indicated in *italics*

Dose: six-month alendronate dosage as one cumulative dose. Both zoledronic acid and denosumab were counted according to the actual number of injection events

For osteoporosis, Zoledronic acid was administered intravenously(I.V.) at a dose of 5 mg per infusion, once annually. For bone metastases from malignant tumor, Zoledronic acid was administered intravenously (I.V.) at a dose of 4 mg per infusion, once 28 days.[Zometa^®^; Novartis Pharma AG,,Basel, Switzerland]

For osteoporosis, Denosumab was administered subcutaneously (S.C.) at a dose of 60 mg per injection,,once every six months. For bone metastases from malignant tumor, Denosumab was administered subcutaneously (S.C.) at a dose of 120 mg per infusion, once every 4 weeks.[Prolia^®^; Amgen Inc.,Thousand Oaks, CA, USA]

Alendronate sodium was administered orally (oral) at a dose of 70 mg, once weekly. [Fosamax^®^; Merck & Co., Inc., Kenilworth, NJ, USA]

Ibandronate was administered intravenously(I.V.) at a dose of 6 mg, once 3–4 weeks. [Bonviva^®^; Roche Holding AG, Basel, Switzerland]

## Results

The clinical characteristics investigated as risk factors for MRONJ are described in Table 2 at the patient level and in Table 3 at the tooth level.

**Table 3:** Summary of the data at a tooth level in LDA and HDA groups

Characteristic		LDA	HDA	*p*-value
Sites of tooth extraction, *n*	Mandible	28	16	0.213
	Maxilla	19	19	
Indications, *n*	Periapical periodontitis	4	3	0.631
	Periodontitis	9	11	
	Fracture teeth	2	1	
	Residual roots or crowns	32	20	
Follow-up duration(months)	Mean ± SD*	7.3 ± 1.39	6.9 ± 0.25	0.424
Time until mocosal healing, *n*	≤ 1 month	46	28	*0.012*
	>1 month	1	7	
Whether developed MRONJ, *n*	Yes	0	2	0.097
	No	47	33	

The *p*-values described under LDA and HDA correspond to those obtained with the Chi-square/Fisher’s exact test or Mann–Whitney U/t test (*) when data were ordinal. Significant *p*-values (*p* ≤ 0.05) are *italicized*

A significantly higher risk of developing MRONJ was observed at the patient level among those with age(*p* = 0.013) 、sex (*p* = 0.001) and primary disease (*p* < 0.001). For LDA group, the primary disease was non-malignant in 31 cases (30 cases of osteoporosis and 1 case of osteogenesis imperfecta), while for HDA group, the primary disease was malignant in 20 cases (including 12 cases of breast cancer, 3 cases of lung cancer, 2 cases of multiple myeloma, and 1 case each of prostate cancer, neuroendocrine cancer, and uterine cancer). Specific ARD used (*p* < 0.001) and time on ARD(dose) (*p* < 0.001) were also show significant differences, although time since last administration of ARD did not show significant differences(*p* = 0.132).

**Fig. 3:**
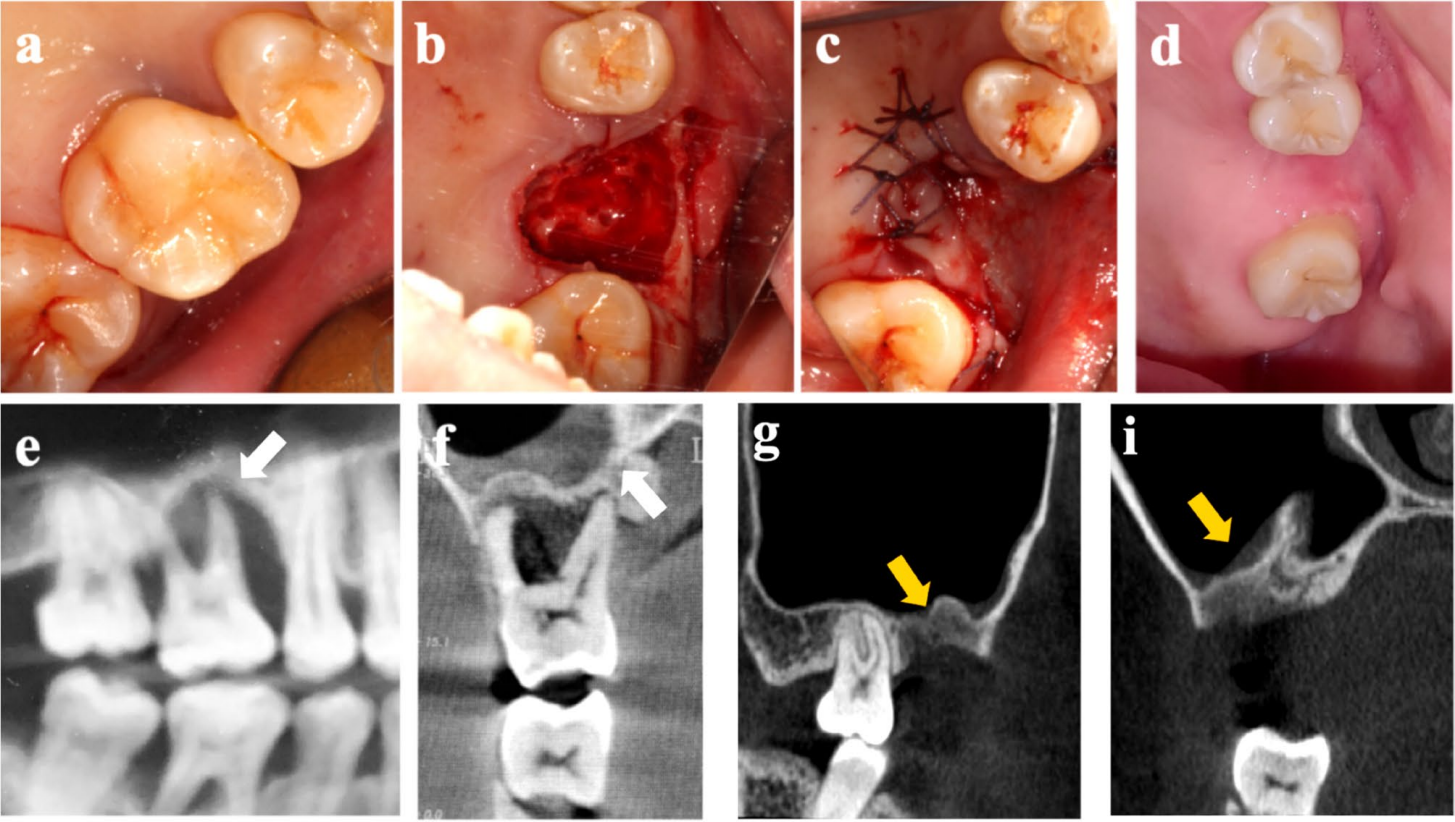
Clinical (**a**, **b**, **c**, **d**) and Imaging examination (**e**, **f**, **g**, **i**) of an 28-year-old female with breast cancer treated with zoledronic acid. Clinically, the maxillary first molar exhibited severe periodontitis, and no evidence of exposed bone was observed during clinical evaluation (**a**), the tooth was extracted using the Mucoperiosteal flap combined with perforation technique (**b**, **c**), and 3 month later, the extraction socket demonstrated complete mucosal healing without clinical signs of infection or inflammation (**d**). In sagittal (**e**) and coronal (**f**) CBCT views, angular bone resorption of the proximal mesial up to the root apex and inflammation of the mucosa at the floor of the maxillary sinus are pointed out by white arrows. CBCT images obtained 3 months postoperatively demonstrated new bone formation at the extraction socket in sagittal (**g**) and substantial regression of inflammation region of the mucosa at the floor of the maxillary sinus in coronal (**i**) CBCT views depicted by yellow arrows

When examining the extraction sites, a total of 82 extraction sites across 51 patients were analyzed. The majority of extraction sites healed successfully. During follow-ups, all extraction sites in the LDA group healed successfully (Fig. 3).However, MRONJ occurred in 2 separate cases within the HDA group (Table 4), resulting in healing rate of 100% (47/47) in LDA group and 94.3% (33/35) in HDA group. Statistical analysis using the Generalized Estimating Equations (GEE) revealed significant differences in mucosal healing outcomes between groups (*p* = 0.012) These findings underscore the importance of differentiation between LDA and HDA groups in healing outcomes.

**Table 4:** Clinical baseline data of patients with MRONJ after tooth extraction

Number	Sex	Age	Primary disease	Systemic disease	Time since last administration of ARD (months)	Time on ARD (dose)	Medicine	Sites of tooth extraction	Indications	Unhealedsites
1	Female	60	Breast cancer	diabetes	6	84	Zoledronicacid+ Anlotinib hydrochloride	15\14\24\25\32\ 42\48	Periodontitis	48
2	Female	65	Brcast cancer	diabetes	8	72	Zoledronic acid	36	Periodontitis	36

Patient 1 had an exposed jaw 1 month after surgery, in patient 2 the mucosa showed obvious redness and swelling 2 months after surgery, and the fistula reached the bone surface.Both patients were diagnosed with MRONJ

According to follow-up, in the HDA group, 3 patients resumed ARD therapy, with the earliest reinstatement occurring at 3 months post-operation. 1 patient experienced breast cancer recurrence at the 6-month follow-up visit (during planned prosthetic evaluation). 5 patients remained clinically stable; after multidisciplinary consultation, a decision was made to discontinue the medication permanently with close monitoring. Unfortunately, effective follow-up information regarding ARD resumption was not available for the remaining patients in this group.For most patients in the LDA group, the medication was switched to alternative regimens such as teriparatide, calcitriol, calcium, or vitamin D supplements post-extraction.

## Discussion

Tooth extraction has been traditionally considered the major local risk factor of MRONJ. The risk of MRONJ in osteoporotic patients undergone tooth extraction ranging from 1%−3.4% [18–22], while in cancer patients the incidence is about 1.6–14.8% [23, 24].However, avoiding extractions due to MRONJ concern is unwarranted, as infection may be the primary cause of osteonecrosis [25]. Persistent chronic inflammation can lead to spontaneous tooth loss, often complicating healing and increasing the risk of developing MRONJ [26–29]. Teeth with severe endodontic and/or periodontal disease, particularly those accompanied by odontogenic infection, when a conservative approach is not possible and a good tooth prognosis is not guaranteed, should be extracted timely [12, 23, 30–32].Thus, we used a new method Mucoperiosteal flap combined with perforation technique for tooth extraction for MRONJ risk patients.Our findings indicate that the new approach significantly reduces the risk of MRONJ after tooth extraction.While for different groups, mucosal healing after tooth extraction is longer in HDA group.

There is currently no consensus on the optimal protocol for extracting teeth in patients at risk of MRONJ. Otto et al. implemented several prophylactic interventions during extractions, which included: (1) long-term antibiotic prophylaxis, (2) minimally invasive extraction techniques, (3) smoothing of sharp bone edges, and (4) tight mucosal closure of the extraction wound. Using these measures, Otto et al. reported that only 4 out of 43 cancer patients treated with bisphosphonate medications (BMAs) developed MRONJ [33]. Similarly, Lodi et al. proposed an integrated prevention strategy, which included: (1) Daily mouth rinsing with 0.2% chlorhexidine; (2) performing professional oral hygiene treatment, including plaque and calculus removal, 2–3 weeks prior to extraction; (3) administering 1 g of amoxicillin every 8 h for 3 days prior to extraction; and (4) clearing and scraping the extraction socket to remove granulation and infected tissues, followed by the creation of a vertical incision and preparation of a coronary flap, which is then advanced to tightly close the wound. In their study, 23 patients treated with ARDs did not develop MRONJ after undergoing tooth extraction with these precautions [34]. These findings have been supported by multiple subsequent studies, which highlight the effectiveness of primary healing of extraction wounds combined with perioperative antibiotic therapy in reducing MRONJ risk [35–37].

Our group’s prior work analyzed the pathological features of MRONJ, identifying three distinct zones in the lesion: an inflammatory zone, an osteosclerotic zone, and a bone reaction zone, from superficial to deep [13]. The inflammatory zone is surrounded by osteosclerotic bone, which often presents as multiple empty bone sockets, indicating poor or absent blood flow. Inspired by techniques in implantology, specifically the use of perforation to increase blood flow in the bone grafting bed, our group designed a modified jaw bone curettage surgical plan using cortical perforation technique. In a small cohort of 10 MRONJ patients, the use of cortical perforation resulted in a 0% recurrence rate, compared to 77.8% recurrence in 18 patients treated with traditional scraping methods [14]. Based on these findings, we introduced a novel approach for tooth extraction, combining the mucoperiosteal flap with perforation, which was tested in a small-sample clinical study and showed a postoperative healing rate of 97% (30/31 extraction sites) [15].

In the present clinical study, with a larger sample size, the postoperative healing rate was 97.6% (80/82 extraction sites). The results showed differences in age and gender distribution between the groups. However, it is well-established in clinical practice that postmenopausal women experience accelerated bone loss—up to three times faster than men—which often leads them to seek treatment more actively. This may partly explain the significant demographic variations observed in our study population.It is noteworthy that MRONJ developed at one extraction site in each of two patients(Table 4). Both cases shared several notable characteristics: (1) the primary diseases were malignant tumors(breast cancer); (2) the patients had received high-dose ARDs (one patient was treated with high-dose ARDs alone, while the other also received concomitant therapy with antiangiogenic agents); (3) radiographically confirmed preoperative odontogenic infection was present at the extraction sites; and (4) the affected extraction sites were located in the mandible. Zhao ning et al. found that patients treated with ARDs and antiangiogenic agents had an advanced clinical stage and a bigger proportion of necrotic jawbone exposure compared to patients treated with ARDs alone [38]. Hongyuan Huang et al. provided new evidence for the vascular mechanisms involved in healing difficulties in MRONJ and highlighted the potential adverse effect of antiangiogenic agents on oral mucosal microcirculation, contributing to impaired wound healing after surgery in MRONJ patients [39]. Our findings showed a significant interaction indicates that the trends in healing probability over time differed significantly between LDA and HDA groups. HDA groups have a longer healing period than LDA groups, despite most of them were healed completely within 1 month.

A comprehensive understanding of the risk factors associated with MRONJ following tooth extraction in patients receiving ARD therapy is essential for clinical decision-making. Risk factors for MRONJ development have been extensively investigated in recent studies. Soutome et al. in their retrospective study involving 189 patients from two institutions, identified independent risk factors for MRONJ. The study highlighted that prolonged use of ARDs and the presence of localized infection significantly increased the likelihood of MRONJ [40]. It is important to note that the risk of MRONJ is significantly higher when signs of infection are present before tooth extraction [5], and the duration of medication plays a pivotal role in determining the likelihood of MRONJ development. Specifically, patients who have been treated with ARDs for eight months or longer exhibit a notably increased risk. Moreover, the mode of medication administration also influences the likelihood of MRONJ, with intravenous administration at high doses associated with a significantly higher risk (risk ratio = 14.6) compared to low-dose oral administration. This was also consistent with the findings of our current study.

As for the Time since last administration of ARD (months), although there was no statistically significant difference between the two groups, we still emphasize that the minimuml time should be no less than one month.However, for some patients, especially HDA groups, ARD are life-saving for them.The timing of resuming treatment is indeed a complex clinical decision. Based on our clinical experience, ARD was typically restarted no earlier than 3 months post-operation, and only after complete and firm soft-tissue closure of the extraction socket was confirmed.Upon resumption, all patients received detailed instructions on intensifying their oral hygiene regimen. This included the use of interdental brushes, dental floss, and oral irrigators to meticulously clean plaque and food debris, particularly from interproximal spaces. Furthermore, they were scheduled for regular clinical and radiographic follow-up examinations every 3 to 6 months to allow for early detection and intervention of any potential complications.

This retrospective design inherently holds limitations compared to prospective studies.,Additionally, the study has some other limitations, including the relatively small sample sizes in HDA group. Notably, a higher incidence of postoperative MRONJ was observed in these groups. To strengthen the evidence base and refine extraction protocols for such patients, future studies with larger sample sizes are essential. This research underscores the need for further investigation to enhance prevention strategies and ensure safe tooth extraction practices in vulnerable patient populations.

In conclusion, Mucoperiosteal flap combined with perforation technique for tooth extraction has yielded positive clinical outcomes in patients at risk for MRONJ. The approach provides a promising protocol for safely managing tooth extractions in this MRONJ risk patients.In addition, a prolonged mucosal healing period is expected in HDA group.

## Data Availability

All data generated or analysed during this study are included in this manuscript.

## Abbreviations

MRONJ: Medication-related osteonecrosis of the jaw
ARD: Anti-resorptive drug
LDA: Low-dose antiresorptive drug
HDA: High-dose antiresorptive drug
